# Outcome of Laparoscopic Versus Vaginal High Uterosacral Ligament Vault Suspension at the Time of Hysterectomy

**DOI:** 10.1007/s00192-024-06033-6

**Published:** 2025-02-07

**Authors:** Sascha Vereeck, James Alexander, Michael Carey, Anna Rosamilia

**Affiliations:** 1 Monash Health, Melbourne, VIC Australia; 2 Royal North Shore Hospital, St Leonards, NSW Australia

**Keywords:** Pelvic reconstructive surgery, Pelvic organ prolapse, Uterosacral ligament suspension, Vaginal hysterectomy, Total laparoscopic hysterectomy

## Abstract

**Introduction and Hypothesis:**

High uterosacral ligament suspension (HUSLS) can be used to treat apical pelvic organ prolapse (POP). This can be performed both vaginally and laparoscopically. Data comparing the two suspension procedures remain limited. The aim of this study is to compare the effectiveness and safety of vaginal HUSLS and laparoscopic HUSLS at the time of hysterectomy.

**Methods:**

This is a retrospective cohort study of women who underwent hysterectomy between 2019 and 2021 at a tertiary urogynaecology unit. Either vaginal or laparoscopic hysterectomy was performed, followed by vaginal or laparoscopic HUSLS respectively. Women were followed up at 6 weeks, and at 6 and 12 months postoperatively. The primary outcome was symptomatic recurrence of vaginal bulge symptoms. Secondary outcomes were anatomical recurrence, re-treatment and safety.

**Results:**

A total of 111 women met the inclusion criteria. Twelve were excluded, leaving 99 for analysis. HUSLS was performed vaginally in 47 and laparoscopically in 52 women. There was no significant difference in demographics between the groups. At 12 months, 92% in the vaginal group and 48% of the laparoscopic group reported no symptoms of prolapse, 67% vs 36% had no anatomical recurrence and 0% vs 34% had re-treatment respectively. Logistic regression (adjusting for age, BMI, menopausal status, preoperative stage apical prolapse, procedure type) demonstrated that the laparoscopic route was the only variable associated with recurrent prolapse at or beyond the hymen and symptomatic prolapse at 12 months.

**Conclusions:**

Symptomatic and anatomical recurrent POP was associated with this technique of laparoscopic HUSLS. Further research should consider prospective evaluation of these or modified techniques.

## Introduction

Pelvic organ prolapse (POP) is a common pelvic floor disorder with a substantial impact on quality of life. Surgery is generally undertaken when significant symptoms of prolapse are demonstrated, with a reported lifetime risk of surgery of 13–19% [1].

A variety of surgical techniques for apical POP have been reported. One treatment option is high uterosacral ligament suspension (HUSLS) [2]. HUSLS is defined as suspension of the vaginal apex to the unilateral or bilateral uterosacral ligament(s) (USL) using sutures. HUSLS was first described as a transvaginal procedure [3]. However, this procedure can also be performed laparoscopically [2]. USL suspension is often performed at the time of a hysterectomy to suspend the apex, in addition to its use during vaginal vault prolapse repair or uterine suspension [3].

A disadvantage of the vaginal approach is the inability to reliably view the ureter prior to suture placement. The ureter lies close to the USL, particularly in its mid and distal sections [2]. Ureteral injury and/or obstruction by kinking is a potential complication of this procedure; therefore, an intraoperative cystoscopy is routinely performed [4]. The advantage of the laparoscopic approach is the superior visualisation of the anatomy and localisation of the ureters, and the ability to incise the peritoneum and reflect the ureters laterally. This would potentially minimise the risk of ureteral injury and obstruction, allowing safe high suture placement [2]. Furthermore, a total laparoscopic hysterectomy with HUSLS for POP may be beneficial; in addition, when bilateral salpingo-oophorectomy (BSO) or salpingectomy (BS) is indicated or preferred.

Although there are potential advantages of the laparoscopic versus the vaginal approach to USL suspension, research regarding the efficacy and safety of this procedure is limited.

A study was performed to compare vaginal with laparoscopic HUSLS at the time of hysterectomy for POP in order to examine the effectiveness and safety of the two procedures.

## Materials and Methods

This is a retrospective cohort study of 111 women who underwent hysterectomy as part of management of POP between 2019 and 2021 at a tertiary urogynaecology unit and private practice. The cohort study included women who presented at the unit with symptomatic POP and wished to have management by hysterectomy and HUSLS. Women who had undergone prior prolapse procedures and those were unable to complete questionnaires owing to language barriers were excluded from this cohort study.

A hysterectomy was performed if the patient presented with at least stage 2 bothersome prolapse and preferred to have a hysterectomy after being informed of all treatment options and/or there was a clinical indication for hysterectomy. Shared decision making, as routinely performed in daily practice, led to either vaginal or laparoscopic hysterectomy being performed; the latter always when BSO was requested for pathological or opportunistic reasons as this was not always feasible vaginally, followed by HUSLS.

Vaginal HUSLS was performed bilaterally using either two delayed absorbable sutures (0 PDS™) and two permanent sutures (2/0 Prolene™) bilaterally, or with four delayed absorbable sutures (0 PDS™).

After performing the vaginal hysterectomy, the USL was identified by putting traction on the previous USL pedicle. One bite of the USL was taken as high and as medial as possible with 0 PDS™. A second suture, either 0 PDS™ or 2/0 Prolene™, was placed on the USL, above the previous suture. The same steps were repeated on the other side. Rectal examination was performed. An anterior vaginal repair was performed before the closure of the vaginal vault. Afterwards, the highest USL suture was attached to the pubocervical fascia and posterior fascia at the vaginal vault. The lower USL sutures were passed through the posterior vaginal vault. Next, the vaginal vault and anterior vaginal wall were closed, the suspension sutures tied under (if Prolene) or over the vaginal epithelium (if PDS), and a cystoscopy checking for ureteric patency was performed at this time. Posterior vaginal repair was performed as necessary.

A laparoscopic hysterectomy was performed if a concomitant BSO or BS was required. First, the USL was identified, and the peritoneum was dissected lateral from the ligament, after identifying the ureter, to reflect the ureter and to prevent ureteric kinking later at the time of the USL suspension. The same step was repeated on the other side. The hysterectomy was completed.

Laparoscopic HUSLS and closure of the vaginal vault was performed with a delayed absorbable barbed suture (0 V-Loc™). Closure of the vault commenced at the right angle. After the vault closure, the suturing was continued toward the left USL. At least three bites, and more if necessary, of the USL were taken, until the most proximal part of the USL was reached. Then, another three bites of the USL towards the vault were taken and incorporated into the vault, suturing until the right angle of the vaginal vault was reached again. Next, the same steps were repeated on the right USL. Both shortened USLs were approximated, incorporating the peritoneum and closure of the pouch of Douglas. Afterwards, anterior and posterior native tissue vaginal repairs were performed as deemed necessary by the surgeon, although it was noted that particularly the anterior repair was limited and difficult to perform owing to the inability to reach the elevated vaginal vault. Cystoscopy and rectal examination were performed at the end of the surgery for assessment of ureteral patency and to exclude rectal injury.

Surgical procedures were performed by consultant or urogynaecology fellows under supervision. Intraoperative and postoperative complications were noted as per Clavien–Dindo classification [5].

All patients underwent a standardised interview and Pelvic Organ Prolapse Quantification System (POP-Q) examination before, during, and after surgery. The POP-Q assessment was performed with Valsalva in the outpatient setting and with mechanical traction intraoperatively. Symptoms of POP were defined as the sensation of a vaginal lump or bulge [6].

Women were followed up at 6 and 12 months postoperatively, and beyond as per normal practice pattern at the time. The latest visit was used for the patients’ > 12 months outcomes. Participants who were only reviewed at 12 months were excluded from the 6-month subanalysis, but not from the overall analysis.

The primary outcome was symptomatic recurrence of POP, defined as the sensation of a vaginal lump or bulge.

Secondary outcomes were anatomical recurrence, measured as any POP of any compartment past the hymen, re-treatment with surgery or pessary and safety based on intraoperative and postoperative complications.

Statistical analysis was carried out with IBM SPSS version 29 (IBM Corporation, Armonk, NY, USA). For nominal data, the Chi-squared test was used to compare outcomes between the different procedures. Continuous data were analysed using a *t* test. Logistic regression was used to assess the correlation between age, BMI, menopausal status, preoperative apical prolapse stage and the type of procedure on the likelihood that participants had any prolapse > 0 and the likelihood of symptomatic prolapse at 12 months. A *p* value of < 0.05, with a 95% confidence interval (CI), was considered statistically significant. This study was approved by the local human research ethics committee (HREC/77947/MonH-2021–273831(v1)).

## Results

Of 111 women, 12 were lost to follow-up postoperatively and excluded from the analysis, leaving 99.

Mean age and BMI at presentation were 64 years (SD 9.9) and 26 kg/m^2^ (SD 3.8) respectively. Vaginal hysterectomy with HUSLS was performed in 47 women, and 52 women underwent laparoscopic hysterectomy with HUSLS. There was no significant difference in demographics and clinical examination pre- and intraoperatively for either of the groups (Table 1).

**Table 1:** Demographics (*N* = 99)

	Vaginal hysterectomy (*n* = 47)	Laparoscopic hysterectomy (*n* = 52)	*p* value
Mean age (years)	66 (SD 9.8)	63 (SD 9.8)	0.172
Mean BMI (kg/m^2^)	26.3 (SD 3.9)	25.9 (SD 3.7)	0.650
Mean parity	3 (SD 1.3)	3 (SD 1.1)	0.650
Postmenopausal	45 (95.7%)	45 (86.5%)	0.112
Use of oestrogen replacement therapy	2 (4.3%)	4 (7.7%)	0.512
Mean preoperative POPQ			
Ba	1.4 (SD 1.7)	1.6 (SD 1.5)	0.715 (95% CI −0.8 to 0.6)
C	−0.8 (SD 2.8)	0.2 (SD 2.7)	0.082 (95% CI −2.2 to 0.1)
Bp	−0.7 (SD 1.6)	− 0.1 (SD 1.5)	0.093 (95% CI −1.2 to 0.1)
Preoperative stage anterior wall prolapse			0.748
0	1 (2.1%)	0 (0%)	
I	2 (4.3%)	3 (5.8%)	
II	20 (42.6%)	22 (42.3%)	
III	24 (51.0%)	27 (51.9%)	
IV	0 (0%)	0 (0%)	
Preoperative stage apical prolapse			0.086
0	0 (0%)	0 (0%)	
I	19 (40.4%)	11 (21.2%)	
II	20 (42.6%)	26 (50.0%)	
III	8 (17.0%)	15 (28.8%)	
IV	0 (0%)	0 (0%)	
Preoperative stage posterior wall prolapse			0.078
0	2 (4.3%)	4 (7.7%)	
I	15 (31.9%)	6 (11.5%)	
II	28 (59.6%)	37 (71.2%)	
III	2 (4.3%)	5 (9.6%)	
IV	0 (0%)	0 (0%)	
Mean intraoperative POP-Q			
Ba	1.8 (SD 1.6)	2.0 (SD 1.5)	0.671 (95% CI −0.7 to 0.5)
C	1.2 (SD 1.2)	1.3 (SD 1.8)	0.744 (95% CI −0.8to 0.6)
Bp	0.4 (SD 1.2)	0.9 (SD 1.1)	0.077 (95% CI −0.9 to 0.1)
Intraoperative stage anterior wall prolapse			0.118
0	0 (0%)	0 (0%)	
I	1 (2.1%)	0 (0%)	
II	20 (42.6%)	16 (30.8%)	
III	26 (55.3%)	36 (69.2%)	
IV	0 (0%)	0 (0%)	
Intraoperative stage apical prolapse			0.675
0	0 (0%)	0 (0%)	
I	2 (4.3%)	3 (5.8%)	
II	29 (61.7%)	28 (53.8%)	
III	16 (34.0%)	21 (40.4%)	
IV	0 (0%)	0 (0%)	
Intraoperative stage posterior wall prolapse			0.160
0	0 (0%)	0 (0%)	
I	1 (2.1%)	0 (0%)	
II	41 (87.2%)	42 (80.8%)	
III	5 (10.6%)	10 (19.2%)	
IV	0 (0%)	0 (0%)	

For the laparoscopic HUSLS, 0 V-Loc™ wound closure device (PDS) was used in all participants. In 32 women (68%), 0-PDS™ was used for the vaginal HUSLS for all four sutures and, in 15 women (32%), a combination of 0-PDS™ and 2–0 Prolene™ bilaterally was used for the vaginal HUSLS.

In the vaginal hysterectomy group, an anterior vaginal repair was performed significantly more often than in the laparoscopic group (100% vs 75%, *p* < 0.001), whereas a posterior repair was performed more frequently in the laparoscopic group (98% vs 91%, *p* < 0.001). In the laparoscopic hysterectomy group, one (1.9%) participant had no additional vaginal repair.

No surgical complications were noted in either group.

Comparing the preoperative and intraoperative POP-Q measurements in the vaginal and laparoscopic hysterectomy groups, mean point Ba, C and Bp were significantly higher intraoperatively. The presence of POP-Q stage 3 prolapse was significantly higher for all the different compartments intraoperatively than preoperatively (*p* < 0.001; Table 2).

**Table 2:** Comparing preoperative and intraoperative Pelvic Organ Prolapse Quantification (POP-Q) measurements in both vaginal and laparoscopic groups (*N* = 99)

	Preoperatively	Intraoperatively	*p* value
Vaginal group—mean POP-Q point			
Ba	1.4 (SD 1.7)	1.8 (SD 1.6)	0.084
C	−0.8 (SD 2.8)	1.2 (SD 1.2)	< 0.001
Bp	−0.7 (SD 1.6)	0.4 (SD 1.2)	< 0.001
Laparoscopic group—mean POP-Q point			
Ba	1.6 (SD 1.5)	2.0 (SD 1.5)	0.003
C	0.2 (SD 2.7)	1.3 (SD 1.8)	< 0.001
Bp	−0.1 (SD 1.5)	0.9 (SD 1.1)	< 0.001
Vaginal group—stage 3 prolapse			
Anterior	51.0%	55.3%	< 0.001
Apical	17.0%	34.0%	< 0.001
Posterior	4.3%	10.6%	< 0.001
Laparoscopic group—stage 3 prolapse			
Anterior	51.9%	69.2%	< 0.001
Apical	28.8%	40.4%	0.003
Posterior	9.6%	19.2%	< 0.001

Mean follow-up was 17 months (SD 8.7). At the 6-month follow-up, 11 patients did not attend their review, leaving 88 women. However, all were reviewed at 1 year postoperatively.

At 12 months and beyond, 18 patients were lost to follow-up, leaving 81 women.

At the 6-month follow-up, symptomatic recurrent prolapse was significantly higher in the laparoscopic hysterectomy and HUSLS group (33.3% vs 4.7%, *p* < 0.001). In particular, the rate of recurrent anterior and/or apical prolapse was significantly higher in the laparoscopic group (68.9% vs 27.9% (*p* < 0.001), and 6.6% vs 0% (*p* < 0.001) respectively). However, there was no difference in recurrent posterior wall prolapse between the two groups (6.7% vs 2.3%, *p* = 0.620; Table 3).

**Table 3:** Follow-up 6 months postoperatively. Symptomatic and clinical difference as seen between vaginal hysterectomy with high uterosacral ligament suspension (HUSLS) and total laparoscopic hysterectomy with HUSLS (*N* = 88)

	Vaginal hysterectomy (*n* = 43)	Laparoscopic hysterectomy (*n* = 45)	*p* value
Symptomatic recurrent prolapse	2 (4.7%)	15 (33.3%)	< 0.001
Anatomical recurrent prolapse (≥ stage II)	14 (32.6%)	21 (46.7%)	0.176
Mean POP-Q			
Ba	−1.7 (SD 1.3)	−0.2 (SD 1.8)	< 0.001 (95% CI −2.2 to 0.8)
C	−7.5 (SD 0.9)	−6.1 (SD 2.9)	0.003 (95% CI −2.3 to −0.5)
Bp	−2.8 (SD 0.6)	−2.7 (SD 0.9)	0.442 (95% CI −0.5 to 0.2)
Stage of anterior wall prolapse			< 0.001
0	15 (34.9%)	6 (13.3%)	
I	16 (37.2%)	8 (17.8%)	
II	12 (27.9%)	24 (53.3%)	
III	0 (0%)	7 (15.6%)	
IV	0 (0%)	0 (0%)	
Stage of apical prolapse			< 0.001
0	43 (100%)	30 (66.7%)	
I	0 (0%)	12 (26.7%)	
II	0 (0%)	1 (2.2%)	
III	0 (0%)	2 (4.4%)	
IV	0 (0%)	0 (0%)	
Stage of posterior wall prolapse			0.620
0	38 (88.4%)	38 (84.4%)	
I	4 (9.3%)	4 (8.9%)	
II	1 (2.3%)	3 (6.7%)	
III	0 (0%)	0 (0%)	
IV	0 (0%)	0 (0%)	

*POP-Q* Pelvic Organ Prolapse Quantification

One year after surgery, there was significantly more symptomatic (52.3% vs 8.1%, *p* < 0.001) and anatomical (63.6% vs 37.8%, *p* = 0.02) recurrent prolapse after laparoscopic hysterectomy with HUSLS compared with the vaginal approach. Recurrent anterior and/or apical prolapse was significantly more prevalent and of a higher stage in the laparoscopic group (*p* < 0.001). However, no significant difference was seen in the posterior compartment (Table 4).

**Table 4:** Follow-up 12 months and more postoperatively. Symptomatic and clinical difference as seen between vaginal hysterectomy with high uterosacral ligament suspension (HUSLS) and total laparoscopic hysterectomy with HUSLS (*N* = 81)

	Vaginal hysterectomy (*n* = 37)	Laparoscopic hysterectomy (*n* = 44)	*p* value
Symptomatic recurrent prolapse	3 (8.1%)	23 (52.3%)	< 0.001
Anatomical recurrent prolapse (≥ stage II)	14 (37.8%)	28 (63.6%)	0.021
Mean POP-Q			
Ba	0.0 (SD 1.7)	0.4 (SD 1.7)	< 0.001 (95% CI −2.2 to −0.7)
C	−6.8 (SD 1.7)	−5.4 (SD 3.4)	0.023 (95% CI −2.7 to −0.2)
Bp	−2.4 (SD 1.6)	−2.7 (SD 0.9)	0.361 (95% CI −0.3 to 0.8)
Stage anterior wall prolapse			0.008
0	8 (21.6%)	4 (9.0%)	
I	11 (29.7%)	4 (9.0%)	
II	16 (43.2%)	26 (59.0%)	
III	2 (5.4%)	10 (22.7%)	
IV	0 (0%)	0 (0%)	
Stage apical prolapse			< 0.001
0	34 (91.9%)	22 (50.0%)	
I	2 (5.4%)	17 (38.6%)	
II	0 (0%)	1 (2.3%)	
III	1 (2.7%)	4 (9.0%)	
IV	0 (0%)	0 (0%)	
Stage posterior wall prolapse			0.447
0	30 (81.1%)	36 (81.1%)	
I	3 (8.1%)	5 (11.4%)	
II	2 (5.4%)	3 (6.8%)	
III	2 (5.4%)	0 (0%)	
IV	0 (0%)	0 (0%)	
Retreatment			< 0.001
Pessary	0 (0%)	11 (25.0%)	
Surgery	0 (0%)	4 (9.0%)	

*POP-Q* Pelvic Organ Prolapse Quantification

At 12 months, in the vaginal group, none of the participants required treatment for recurrent prolapse, whereas in the laparoscopic group 15 (34%) required retreatment (*p* < 0.001) for recurrent bothersome POP. Of these, 11 women (25%) underwent pessary placement and 4 (9% opted for repeat pelvic reconstructive surgery.

Binomial logistic regression was performed to ascertain the effects of age, BMI, menopausal status, preoperative apical stage and the type of procedure on the likelihood that participants had any prolapse > 0 and the likelihood of symptomatic prolapse at 12 months. Only the laparoscopic procedure showed an association with recurrent prolapse beyond the hymen and symptomatic prolapse.

## Discussion

In this study cohort and using the described surgical techniques, HUSLS at the time of hysterectomy was less effective when performed laparoscopically than vaginally. One year after surgery, there was significantly more symptomatic and anatomical recurrent prolapse after laparoscopic hysterectomy with HUSLS than after the vaginal approach. Furthermore, if recurrent prolapse was present, it was a higher stage prolapse in the laparoscopic group.

No complications were noted in this cohort of patients. This is analogous to previous studies [2, 7, 8]. Although the laparoscopic approach is thought to be the safer route owing to better visibility and therefore less ureteric injury, the meta-analysis of Ronsini et al. showed a non-significantly higher risk for both minor and major complications than the vaginal approach [9].

Intraoperative POP-Q measurements and prolapse stages were significantly higher than preoperatively, both in the vaginal and the laparoscopic hysterectomy groups. These findings are similar to those of other studies, showing that intraoperative evaluation of the prolapse by mechanical traction under anaesthesia often results in a greater degree of descent, especially in the apical and posterior compartments [10–12]. Based on the intraoperative findings, the extent of the surgical procedure may change and the patient should be counselled beforehand of this possibility.

The systematic review of Vermeulen et al. presents an overview of the current literature on laparoscopic USL suspension as a treatment for apical POP. Their pooled anatomical success rate (defined as the total number of women cured of apical prolapse ≥ stage 2) was 90% for all laparoscopic uterosacral suspension procedures (95% CI 83.3–95.5), with a mean follow-up of 22 months [2]. This is similar to our data, which showed an anatomical apical success rate (apical prolapse < stage 2) after laparoscopic HUSLS of 88.7% at 1 year after surgery.

However, Vermeulen et al. showed a pooled subjective cure rate (defined as either satisfaction on the Patient Global Impression of Improvement [PGI-I] scale or the absence of POP symptoms) of 90.5% (95% CI 81.9–96.5) [2], which could not be confirmed in our study (47.7%). We did not measure the PGI-I scale, but compared POP symptoms. The main difference between the studies is that most patients in the study by Vermeulen et al. were treated for mild apical POP, compared with our population with a higher proportion of stages 2 and 3 POP [2].

Douligeris et al. showed in their systematic review and meta-analysis that laparoscopic USL suspension was associated with lower objective (OR 0.47; 95% CI 0.23–0.97; *p* = 0.04) and subjective recurrence rates (OR 0.46; 95% CI 0.23–0.92; *p* = 0.03) than the vaginal approach [13], which is opposite to our data. However, their study showed no significant difference in re-treatment rate [13]. Furthermore, it is important to note that in their study, the vaginal group had more stage 3 and 4 prolapse preoperatively (48.62%, 229 out of 471 patients) than the laparoscopic group (27.14%, 92 out of 339 patients) [13]. The results should be interpreted with caution as the meta-analysis is based on retrospective studies with small sample sizes and short-term follow-up.

In contrast, the meta-analysis of Ronsini et al. showed no difference in recurrence rate and reoperation rate between vaginal and laparoscopic HUSLS [9]. This is similar to other studies showing no significant difference in subjective and anatomical outcomes 6–12 months after vaginal HUSLS vs robot-assisted or laparoscopic HUSLS at the time of hysterectomy [7–9, 14].

The surgical technique of the laparoscopic HUSLS was reviewed by Vermeulen et al. However, no differences in outcome were detected based on type and number of sutures. Thus, no recommendations could be given regarding the best technique in their review [2].

There are several differences between the laparoscopic and vaginal approaches in this study that might explain the different outcome. First, in the laparoscopic group, one continuous delayed absorbable suture was utilised versus four separate sutures (68% had delayed absorbable sutures) in the vaginal group. Other studies could not demonstrate any difference in surgical success rate after vaginal HUSLS with absorbable versus permanent sutures [15–18]. Second, it is the authors’ opinion that during vaginal USL suture placement, the depth of purchase of tissue often includes part of the underlying iliococcygeus muscle, whereas laparoscopic suture placement, although higher, does not as often achieve the same depth.

It was noted that the adequacy of the anterior vaginal wall repair was different when performed after a vaginal approach (up to the apex and up to the level of the uterosacral pedicles), as the suspension sutures are not at that point tied. This is compared with a very limited anterior vaginal repair after the laparoscopic hysterectomy and uterosacral vault suspension that is difficult to perform. In addition, during the vaginal approach, the vaginal USL sutures are attached to the pubocervical fascia at its most proximal level. This does not occur with the laparoscopic approach as the vaginal vault is closed.

Future studies could consider performing an anterior vaginal repair prior to laparoscopic hysterectomy with USL suspension, and attaching the suspension sutures to the repair.

The limitations of this study include that it is a retrospective analysis of a patient series and results may not be applicable to the general population or utilising other laparoscopic or vaginal surgical techniques. The strengths of this study are a follow-up of at least 1 year and demographically equivalent groups to minimise selection bias.

To the best of our knowledge, no long-term data regarding the efficacy of laparoscopic HUSLS exist [2, 9]. Most studies in the literature are retrospective and consist of a small sample size [2, 7–9, 13, 19]. Current literature shows heterogenous data with often no distinction between laparoscopic HUSLS with or without hysterectomy [9, 19, 20].

Ideally, a prospective randomised controlled trial should be performed with long-term follow-up to examine the efficacy and safety of both techniques, including a cost-effectiveness analysis. In this context, the authors would modify their laparoscopic technique to attempt to achieve better outcomes than outlined in this study. This may include adding a non-absorbable suture, addressing the depth of laparoscopic suture purchase and the number of sutures, consideration of performing the anterior vaginal repair prior to the hysterectomy and suspension, and connecting the suspension sutures to the fascial repair.

More research is needed regarding the different surgical techniques of laparoscopic HUSLS to ameliorate surgical and patient-reported outcomes. Furthermore, it is important to determine the place of laparoscopic HUSLS for apical prolapse repair compared with other techniques, especially with greater numbers of hysterectomies performed with laparoscopic or robotic assistance than by using the vaginal approach, including those with the indication of POP.

Houlihan et al. showed that completed adnexal surgery rates are significantly higher in the laparoscopic group than with the vaginal approach and therefore, this could influence surgical decision making [21]. However, because vaginal natural orifice transluminal endoscopic surgery (vNOTES) is becoming more widely available, this makes it feasible to adequately visualise and safely remove the tubes and/or ovaries vaginally.

Another interesting future research project would be to examine vaginal HUSLS versus vNOTES HUSLS, as to date no comparative studies have been made available.

In conclusion, using the surgical techniques described in this study, vaginal hysterectomy with HUSLS and repair was associated with lower rates of objective and subjective recurrent POP than laparoscopic hysterectomy with HUSLS and repair in women with stage 2 and 3 POP.
